# Effective neuronal refractoriness dominates the statistics of superimposed spike trains

**DOI:** 10.1186/1471-2202-12-S1-P273

**Published:** 2011-07-18

**Authors:** Moritz Deger, Moritz Helias, Clemens Boucsein, Stefan Rotter

**Affiliations:** 1 Bernstein Center Freiburg & Faculty of Biology, Albert-Ludwig University, 79104 Freiburg, Germany; 2 Laboratory for Computational Neurophysics, RIKEN Brain Science Institute, Wako City, Saitama 351-0198, Japan

## 

**Figure 1:**
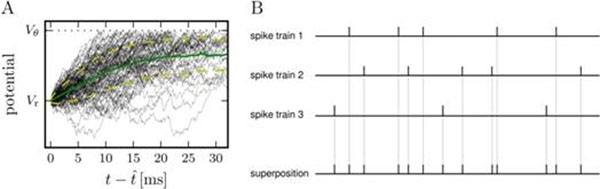
A: Membrane potential trajectories of a simulated neocortical neuron. After each spike, the potential has to charge up until spikes can be initiated by input fluctuations, leading to an effective refractoriness. Green line shows the mean subthreshold trajectory, yellow lines show mean +/- standard deviation. B: Scheme of the independent superposition of three spike trains. Adapted from [6].

The pooled spike trains of populations of neurons are typically modeled as Poisson processes [2]. It is known, though, that the superposition of point processes is a Poisson process if and only if all components are Poisson processes [3]. However, neocortical neurons spike more regularly [1]. Partly this is because they often have a refractory period, but also because the membrane potential is hyperpolarized after each spike, as illustrated in Figure 1A. Here we analyze neuronal spike trains recorded intracellularly *in vivo* from rat somatosensory cortex. We match them with a Poisson process with dead-time [4], which is the simplest model of neuronal activity that incorporates refractory effects. The dead-time here models the effective refractoriness of the neuron, which can be larger than the refractory period due to channel kinetics alone. From the spike train recordings we construct independent superpositions (see Figure 1B) and compare their statistics to our analytical results for the model processes. We find that the effective refractoriness of the neurons dominates the second-order statistics of the superposition spike trains. We uncover profound statistical differences as compared to Poisson processes, which considerably affect the dynamics of the membrane potential of neurons that receive such superpositions, as we further show in numerical simulations (see also [5]).
